# Effects of autologous platelet-rich plasma in recurrent implantation failure: a systematic review and meta-analysis

**DOI:** 10.3389/fendo.2026.1730259

**Published:** 2026-04-14

**Authors:** Zhuo Pan, Xiaoni Guo, Qing Su, Hong Ye

**Affiliations:** 1 Center for Reproductive Medicine, Chongqing Key Laboratory of Human Embryo Engineering, Chongqing Reproduction Genetics Institute, Chongqing Health Center for Women and Children, Women and Children’s Hospital of Chongqing Medical University, Chongqing, China; 2 Department of Obstetrics and Gynecology, Chongqing University Central Hospital & Chongqing Emergency Medical Center, Chongqing, China

**Keywords:** blastocyst, fresh embryo, frozen embryo, *in vitro* fertilization, meta-analysis, platelet-rich plasma, pregnancy, repeated implantation failure

## Abstract

**Objective:**

The role of intrauterine PRP infusion in managing recurrent implantation failure (RIF) remains controversial despite its emerging clinical use. This systematic review aims to evaluate its therapeutic potential in RIF patients and further to investigate variations in outcomes based on transfer cycle type, embryo developmental stage, RIF diagnostic criteria, and endometrial thickness.

**Methods:**

We systematically searched MEDLINE, Embase, the Cochrane Central Register of Controlled Trials, Scopus, and Web of Science for randomized controlled trials (RCTs) investigating PRP treatment for RIF patients from the beginning of the database to May 2025.

**Results:**

This meta-analysis showed that PRP administration significantly improved clinical pregnancy rate (CPR) [OR = 3.18, 95%CI (2.45, 4.14), I^2^ = 3%], biochemical pregnancy rate (BPR) [OR = 2.84, 95%CI (2.22, 3.63), I^2^ = 0%], ongoing pregnancy rate (OPR) [OR = 3.41, 95%CI (2.08, 5.60), I^2^ = 30%] and live birth rate (LBR) [OR=5.10, 95%CI (1.95, 13.37), I^2^ = 75%] in women with RIF. However, PRP intrauterine infusion did not reduce miscarriage rate (MR). Notably, the preterm birth rate was significantly higher in the PRP group compared to controls [OR = 8.24, 95%CI (2.09, 32.41), I^2^ = 0%]. Subgroup analysis demonstrated that PRP improved CPR, BPR and LBR in both the fresh and frozen embryo transfer cycles. Additionally, while PRP increased CPR, LBR and reduced MR in blastocyst transfers [CPR OR = 3.84, 95%CI (2.82, 5.23), I^2^ = 0%; LBR OR = 7.32, 95%CI (3.17, 16.90), I^2^ = 63%; MR OR = 0.27, 95%CI (0.07, 0.96), I^2^ = 54%], these effects were not observed in cleavage-stage embryo transfers. Moreover, PRP administration associated with a higher CPR [OR = 3.84, 95%CI (2.82, 5.23), I^2^ = 0%], OPR[OR = 4.13, 95%CI (1.79, 9.56), I^2^ = 48%], LBR [OR = 7.32, 95%CI (3.17, 16.90), I^2^ = 63%] and a lower MR [OR = 0.27, 95%CI (0.07, 0.96), I^2^ = 54%] in women with ≥3 prior implantation failure, it did not confer the same benefit to those with a history of ≥2 failed cycles.

**Conclusion:**

These findings suggest a possible beneficial role for PRP on pregnancy outcomes to some extent in women with RIF, particularly in cases with ≥3 prior failed transfers, and blastocyst transfer may increase LBR and reduce miscarriage risk. However, further investigation is warranted to determine whether this treatment may pose an increased risk of preterm birth.

**Systematic review registration:**

https://www.crd.york.ac.uk/prospero/, identifier CRD420251061511.

## Key points

Question/objective: What is the impact of platelet-rich plasma (PRP) on repeated implantation failure (RIF) patient, and is this effect influenced by the type of transfer cycle, the stage of the embryos transferred, RIF diagnostic criteria, or the endometrial thickness?

Findings: This study demonstrates that intrauterine infusion of PRP may improve reproductive outcomes in patients with RIF, with similar efficacy observed in both fresh and frozen embryo transfer cycles. Among women with a history of more than three prior implantation failures, PRP administration was associated with higher clinical pregnancy rate (CPR), ongoing pregnancy rate (OPR), live birth rate (LBR), and a lower miscarriage rate (MR). However, this effect was not observed in women with two or more prior failed cycles. Furthermore, the beneficial effects of PRP on CPR and MR were observed only in cycles involving blastocyst transfer. Notably, the preterm birth rate was significantly higher in the PRP group compared to the control group.

Meaning: PRP may confer pregnancy benefits in women with RIF, particularly those with ≥3 prior failures, and blastocyst transfer appears to increase LBR and reduce miscarriage risk. However, safety concerns such as preterm birth warrant attention.

## Introduction

Repeated implantation failure (RIF) refers to the condition in which good-quality embryos fail to implant after multiple transfers (1). With an estimated incidence of 10%, approximately 50% of RIF cases remain unexplained (2). Despite significant advances in assisted reproductive technologies (ART), RIF continues to represent a persistent and clinically challenging problem in reproductive medicine, causing substantial psychological for patients and therapeutic dilemmas for clinicians. This underscores the critical need for developing effective treatments to improve pregnancy outcomes. The clinical management of RIF is further complicated by the absence of standardized diagnostic criteria, resulting in significant heterogeneity in both diagnosis and treatment approaches. Currently, no consensus exists regarding optimal management strategies.

Possible reasons for RIF include impaired endometrial receptivity, poor embryo quality, immune dysregulation, and mismatched coordination between the developing fetus and endometrium. It is estimated that embryonic factors account for approximately one-third of implantation failures, while suboptimal endometrial receptivity and aberrant embryo-endometrial cross-talk contribute to the remaining two-thirds (3, 4). To address these challenges, several interventions aimed at enhancing endometrial receptivity have been proposed, including: endometrial injury (5), intravenous atosiban (6), growth hormone supplementation (7), intrauterine human chorionic gonadotrophin (hCG) administration (8), granulocyte colony-stimulating factor (G-CSF), delivered subcutaneously or intrauterinely (9, 10), and intrauterine administration of peripheral blood mononuclear cells (PBMCs) (11). Despite these advances, RIF remains a leading cause of ART failure. Thus, developing safer and more effective treatments to improve pregnancy outcomes in affected couples remains a critical unmet need.

Autologous platelet-rich plasma (PRP) is a concentrated preparation of platelets derived from whole blood. As an economical source of growth factors, PRP provides high concentrations of vascular endothelial growth factor (VEGF), transforming growth factor-β (TGF-β), and platelet-derived growth factor (PDGF) through α-granule release upon platelet activation (12). These bioactive factors collectively promote tissue regeneration by: stimulating angiogenesis, enhancing cellular proliferation and differentiation, and modulating inflammatory responses (13). The therapeutic potential of PRP has been well-documented in various clinical applications, including wound management, regenerative medicine, and tissue engineering (14).

The human endometrium expresses multiple receptors for growth factors, adhesion molecules, and cytokines that mediate endometrial-embryonic crosstalk during implantation (15). Intrauterine administration of autologous PRP delivers concentrated growth factors and cytokines that enhance endometrial receptivity by modulating the endometrial molecular microenvironment, promoting vascular remodeling, and optimizing the implantation niche. This therapeutic approach creates a more favorable endometrial environment for embryo attachment and subsequent development (16, 17).

Since its first successful application in 2015 for improving refractory endometrium in women undergoing IVF (18), PRP has been investigated in multiple case series and clinical trials, with largely promising outcomes (19, 20). In recent years, intrauterine infusion of PRP has emerged as a focus of research for women with RIF. However, study results remain inconsistent. Several studies report that intrauterine administration of PRP enhances embryo implantation by increasing endometrial thickness in RIF women (21, 22). Enatsu et al. further observed improved pregnancy rates after PRP administration in RIF patients, regardless of endometrial thickness, though PRP did not outperform conventional therapies in augmenting endometrial growth (23). Conversely, other studies have failed to demonstrate PRP’s efficacy as an adjuvant treatment for RIF patients with normal endometrial thickness undergoing embryo transfer (24, 25).

Robust evidence synthesis is critical to inform clinical decision-making for women with RIF. While existing meta-analyses show improved pregnancy rates with intrauterine PRP; however, the heterogeneity in RIF diagnostic criteria (≥2 vs. ≥3 implantation failures) across original studies, along with methodological limitations, compromises their internal validity and preclude definitive conclusions (26, 27). To address this gap, we systematically reviewed randomized controlled trials assessing PRP efficacy in this distinct population, with subgroup analyses stratified by transfer cycle type (fresh vs. Frozen embryo transfer) and embryo developmental stage (cleavage-stage embryo vs. blastocyst), RIF diagnostic criteria (≥2 vs. ≥3 implantation failures), and endometrial thickness (<7mm vs. ≥7mm).

## Methods

This systematic review adheres to the Preferred Reporting Items for Systematic Reviews and Meta-Analyses (PRISMA) and was registered with the International Prospective Register of Systematic Reviews (PROSPERO), number CRD420251061511.

### Search strategy and eligibility criteria

We systematically searched the following databases: MEDLINE, Embase, the Cochrane Central Register of Controlled Trials, Scopus, and Web of Science from the beginning of the database to May 2025. Our search strategy incorporated both free-text terms and MeSH including: “Platelet-Rich Plasma”, “PRP”, “recurrent implantation failure”, “embryo implantation”, “*in vitro* fertilization”, “IVF”, and “intracytoplasmic sperm injection”. We also manually searched references and citations of included studies to identify additional eligible literature.

We included randomized controlled trials (RCTs) that evaluated the efficacy of intrauterine PRP administration versus no intervention, placebo, or other active treatments in improving pregnancy outcomes. We selected eligible studies based on the following criteria: participants were subfertile women with RIF; outcomes included medically confirmed pregnancy outcomes: clinical pregnancy rate (CPR), ongoing pregnancy rate (OPR), live birth rate (LBR), and miscarriage rate (MR). Exclusion criteria included: non-randomized study designs (observation studies, animal studies, case reports, self-pro-post studies); conference abstracts and review articles; and non-English publications. For studies with overlapping populations, we included the publications with the most comprehensive data and largest sample size.

Two independent investigators screened study titles and abstracts, retrieving full-text articles for all records meeting the inclusion criteria. Final eligibility was determined by investigator consensus, with unresolved disagreements adjudicated by a third reviewer.

### Data extraction and assessment of risk bias

Two reviewers independently extracted the following data from each included study using a specifically designed form: study design, sample size; participant characteristics, number of prior implantation failures, transfer cycle type, embryo development stage, intervention details, and outcome measures. All extractions were performed directly into pre-designed standardized electronic form to ensure consistency.

Two independent reviewers evaluated the methodological quality of included studies using the Cochrane risk of bias tool, which covers seven specific domains. Disagreements were resolved through consensus or, when necessary, by consultation with a third reviewer. For each domain, we assigned risk-of-bias judgments (low, high, or unclear) to individual studies.

### Statistical analysis

Odd ratios (ORs) with corresponding 95% confidence intervals (CIs) were calculated for all dichotomous outcomes. The Mantel-Hansel fixed-effects model was used to pool ORs when heterogeneity was absent (I^2^<50%); otherwise, a random-effects model was employed. We assessed potential publication bias using Egger’s test for funnel plot asymmetry, with a *P* < 0.1 considered statistically significant. We performed prespecified subgroup analyses to determine the effect of PRP infusion on pregnancy outcomes, taking into account relevant study characteristics such as transfer cycle (fresh versus frozen), embryo stage (cleavage-stage embryo or blastocyst), RIF diagnostic criteria (≥2 vs. ≥3 implantation failures), and endometrial thickness (<7mm vs. ≥7mm). Sensitivity analyses were conducted to assess the robustness of our conclusions. Data were analyzed using Revman5.3.3 (Cochrane Collaboration, Copenhagen, Denmark, 2014) and Stata 14.0 (StataCorp LP, Texas, USA, 1985-2015), with two-tailed α=0.05.

## Results

### Search results

**Figure 1:**
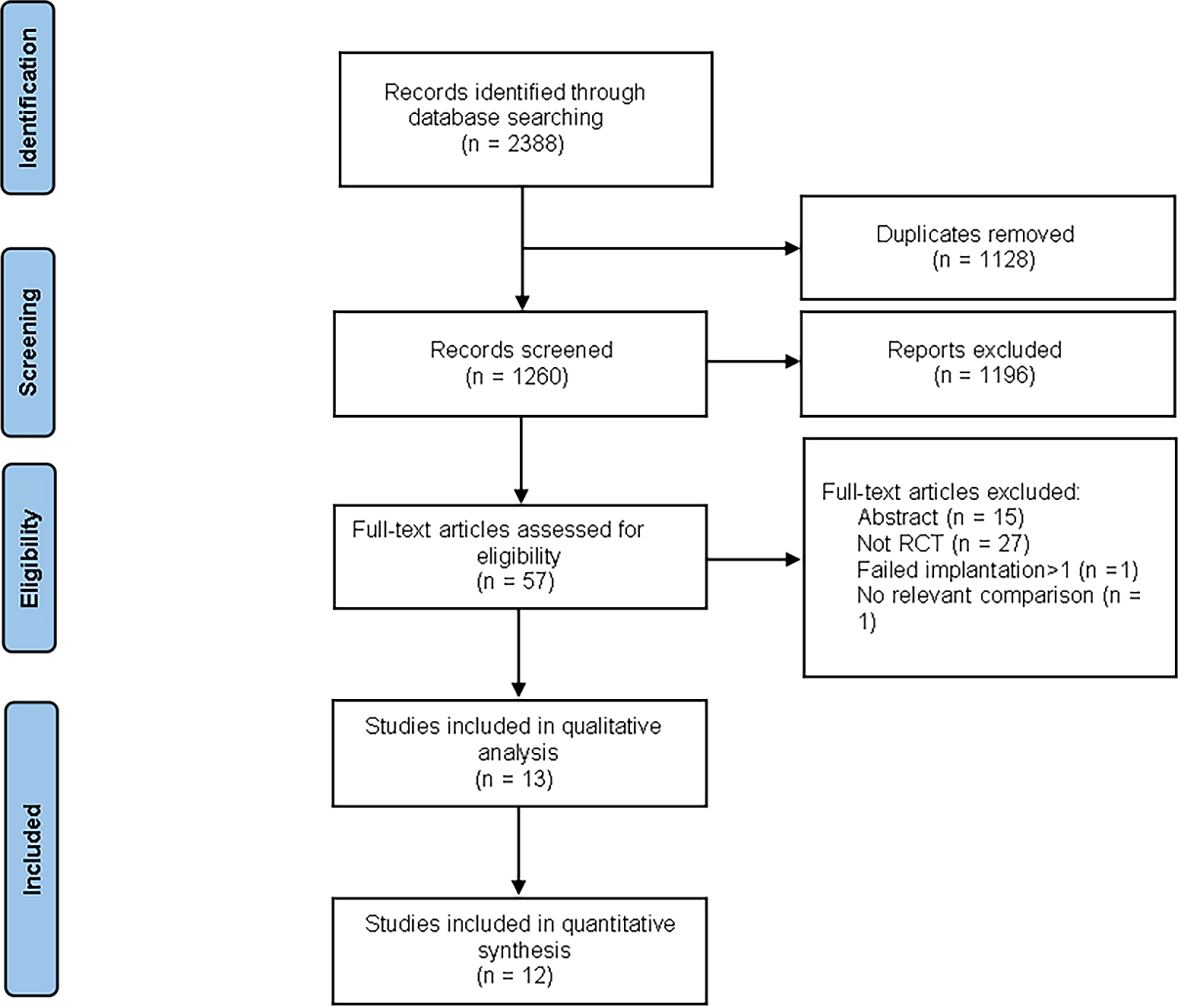
Flow diagram for study selection process.

Our systematic search identified 2388 records. After duplicate removal (n=1128), we screened 1260 unique studies by title/abstract, excluding 1196 irrelevant publications. Full-text review of 57 articles led to exclusion of 44 studies for: conference abstracts (n=15); non-RCT designs (n=27); not part of the literature about recurrent implantation failure (n=1); lack of relevant comparator (n=1). Thirteen studies met all eligibility criteria (24, 28–38), of which twelve provided sufficient data for meta-analysis (24, 28–36, 38). **Figure 1** presents the PRISMA flow diagram.

### Description of included studies

The meta-analysis included 1438 participants (intervention group: n=717; control group: n=721), with a mean age range of 24.9 ± 2.9 to 37.3 ± 6.4 years. Among the included studies: eleven compared PRP intervention versus control (no treatment/placebo) (24, 28–35, 38, 46); one three-arm study evaluated PRP versus control group versus PBMCs (36). Two studies analyzed fresh embryo transfer cycles (29, 36), eight examined frozen cycles (24, 28, 30–35), and two study incorporated both transfer protocols (38, 46). Eight studies performed blastocyst-stage transfers (28–34, 38), while four studies utilized cleavage-stage embryo transfers (24, 35, 36, 46). All participants maintained endometrial thicknesses greater than 7mm. Detailed study characteristics are presented in **Table 1**.

**Table 1:** Characteristics of included trials.

Author /Year	Study design	Sample sizes	participant characteristics	ET cycle	embryo developmental stage	Intervention group	Control group	Outcomes
Nazari 2020 (28)	RCT	97	RIF: failed to conceive after three or more ET with high-quality embryos; age below 40 years old; BMI below 30 kg/m2	Frozen cycle	good quality blastocysts	intrauterine infusion of 0.5ml PRP 48h before ET	did not receive any additional treatment before the ET.	CPR, BPR
Rageh 2020 (29)	RCT	150	RIF: failed to conceive after three or more ET with high-quality embryos; age below 40 years old; BMI below 30 kg/m2	Fresh cycle	good quality blastocysts (Grade A or B)	intrauterine infusion of 0.5-1ml PRP 48h before ET	did not receive any additional treatment before the ET.	BPR
Zamaniyan 2021 (30)	RCT	120	RIF: failed to conceive after three or more ET with high-quality embryos; age between 20–40 years; BMI below 30 kg/m2	Frozen cycle	one or two good quality blastocysts (Grade A or B)	intrauterine infusion of 0.5ml PRP 48h before ET	did not receive any additional treatment before the ET.	CPR, BPR, OPR, MR, IR,
Bakhsh 2022 (31)	RCT	100	RIF: failed to conceive after three or more ET with at least four high-quality embryos; age below 40 years old; BMI below 30 kg/m2	Frozen cycle	good quality blastocysts (Grade A or B)	intrauterine infusion of 0.5ml PRP 48h before ET	only the catheter was transferred and removed without injection or any other action	CPR, BPR
Baybordi 2022 (32)	RCT	94	RIF	Frozen cycle	blastocysts	intrauterine infusion of 0.5-1ml PRP on day 10th of the HRT cycle, performed once or twice per cycle, after 48h of PRP injection, the embryos were transferred	did not receive any additional treatment before the ET	CPR, BPR, OPR, LBR, MR
Ershadi 2022 (24)	RCT	85	a history of two to three IVF failures; age below 40 years old	Frozen cycle	two to three good-quality eight cell embryos (grade A or B based on the embryological score)	intrauterine infusion of 0.5ml PRP 48h before ET	did not receive any additional treatment before the ET.	CPR, BPR, MR
Nazari 2022 (33)	RCT	418	RIF: failed to conceive after three or more ET with high-quality embryos; age between 18–38 years; BMI below 30 kg/m2; FSH level ≤10 mIU/ml	Frozen cycle	one or two good quality blastocysts	intrauterine infusion of 0.5ml PRP 48h before ET	did not receive any additional treatment before the ET.	CPR, BPR, LBR, MR, EPR, MPR
Safdarian 2022 (34)	RCT	120	RIF: failed to conceive after three or more ET with highquality embryos and had at least one frozen good-quality blastocyst-stage embryo; age between 20-40 years; BMI below 30 kg/m2;	Frozen cycle	one to three good-quality blastocyst(s) (Grade A or B)	intrauterine infusion of 0.5ml PRP 48h before ET	did not receive any additional treatment before the ET.	CPR, BPR, OPR, LBR, MR, IR, MPR, Preterm birth rate
Eftekhar 2024 (35)	RCT	72	RIF: with a history of two or more implantation failures; age between 18-42 years;	Frozen cycle	one or two cleavage embryos	intrauterine infusion of 0.5-1ml PRP 48h before ET	did not receive any additional treatment before the ET.	CPR, BPR, OPR, IR
Fazaeli 2024 (36)	RCT	96	RIF: at least 2 RIF in IVF/ICSI; BMI below 30 kg/m2;age below 40 years old	Fresh cycle	the two best day 3 embryos	intrauterine infusion of 0.5-1ml PRP 48h before ET	intrauterine infusion of 0.5ml PBMCs 2 days before ET; did not receive any additional treatment before the ET.	CPR, BPR
Mehrafza 2024 (37)	RCT	200	RIF: failed to conceive after two or more ET with high-quality embryos; age below 41 years old	Frozen cycle	not mentioned	intrauterine infusion of 1ml PRP 48h before ET	intrauterine infusion of 1 ml of G-CSF on the first day of progesterone treatment	CPR, BPR, OPR
Yahyaei 2024 (38)	RCT	69	RIF: failed to conceive after transfer of at least 4 good-quality embryos in a minimum of 3 fresh or frozen ET cycles; history of at least one blastocyst ET; age below 40 years old; BMI between 19-29 kg/m2	Fresh cycle, Frozen cycle	two to three blastocysts	intrauterine infusion of 0.8-1ml PRP 48h before ET	did not receive any additional treatment before the ET.	CPR, LBR, MR, Preterm birth rate
Zargar 2021	RCT	80	RIF: with at least two IVF failures; age below 41 years old.	Fresh cycle, Frozen cycle	one or two embryos	intrauterine infusion of 1.5ml PRP 48h before ET	did not receive any additional treatment before the ET.	BPR, MR, LBR

Only one study compared the effects of intrauterine PRP infusion and G-CSF in patients with RIF (37), and due to insufficient data in the G-CSF group, quantitative analysis could not be performed. Mehrafza et al. reported significantly elevated CPR, BPR, and OPR in the PRP group versus the G-CSF group. The authors concluded that intrauterine infusion of PRP demonstrates superior efficacy to G-CSF for improving reproductive outcomes in RIF (37). The relative efficacy of PRP versus G-CSF in RIF patients remains to be determined in future investigations.

No adverse effects related to PRP administration were reported in any included studies.

### Risk of bias in included studies

**Figure 2:**
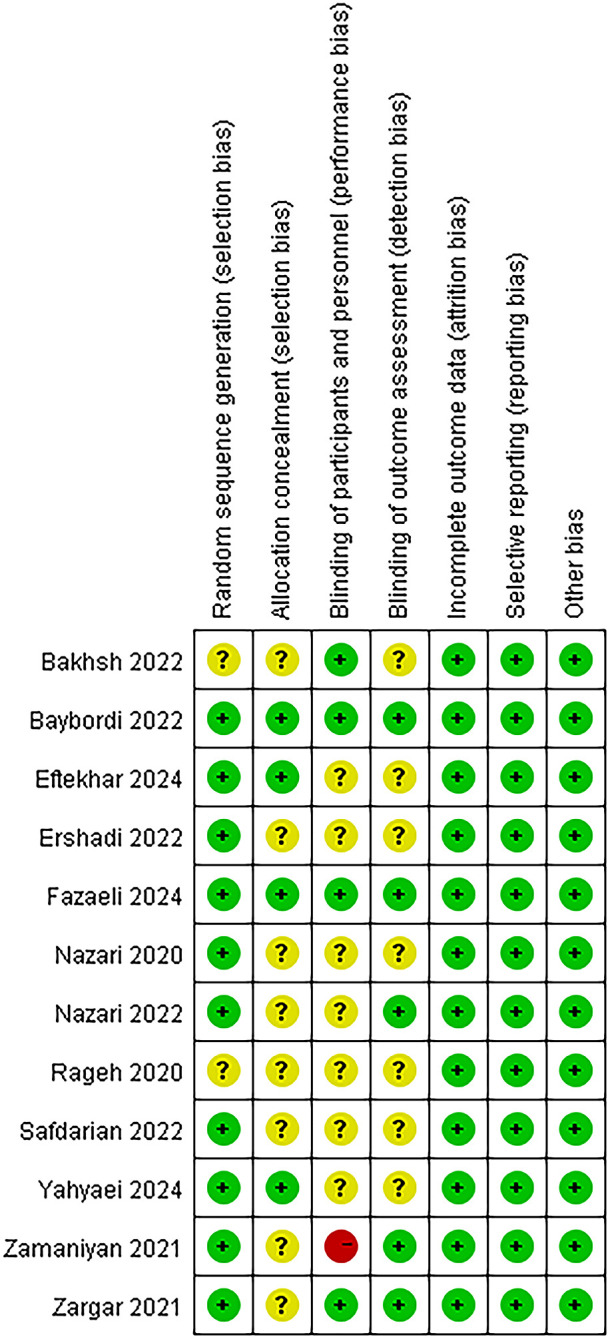
Risk of bias summary: review authors’ judgements about each risk of bias item for each included study.

We assessed the risk of bias in all included studies, as shown in **Figure 2**. Ten studies reported adequate methods for random sequence generation (24, 28, 30, 32–36, 38, 46). Eight studies did not mention allocation concealment and were assessed as having an unclear risk of bias (24, 28–31, 33, 34, 46). No attrition bias was detected in any of the studies. Two studies were rated as having a low risk of bias (32, 36), and one was judged to be at high risk because the participants were not blinded (30).

### Primary outcomes

#### CPR

**Figure 3:**
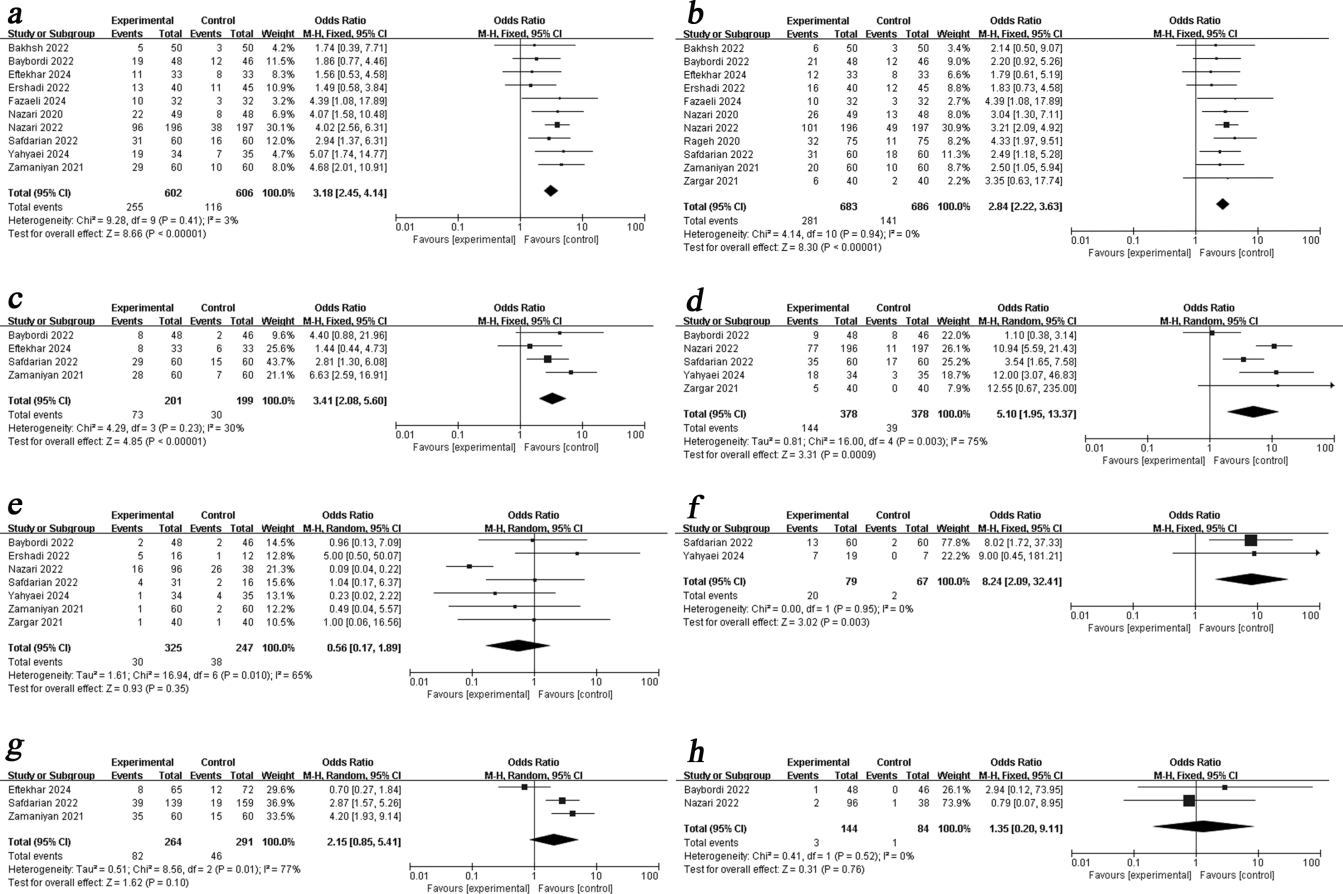
Forest plot showing individual and combined effect size estimates and 95% confidence intervals (CIs) in studies that evaluated the effect of PRP on pregnancy outcomes in women with RIF. **(a)** clinical pregnancy rate. **(b)** biochemical pregnancy rate. **(c)** ongoing pregnancy rate. **(d)** live birth rate. **(e)** miscarriage rate. **(f)** Pre-term rate. **(g)** implantation rate. **(h)** ectopic pregnancy rate.

Ten studies comparing the PRP group with the control group reported CPR. Given the low heterogeneity (I^2^ = 3%), a fixed-effects model was used for the meta-analysis. The results demonstrated that PRP significantly improved CPR [OR = 3.18, 95%CI (2.45, 4.14)] compared to the control group (**Figure 3a**).

#### BPR

Eleven studies compared BPR between the two groups after different interventions. As shown in **Figures 3b**, the pooled results demonstrated higher BPR in the PRP group than in the control group [OR = 2.84, 95%CI (2.22, 3.63)], with no heterogeneity observed (I^2^ = 0%).

#### OPR

Four studies reported OPR, including a total of 400 patients, 201 of whom were in the study group and 199 of whom were in the control group. Using a fixed-effects model for data synthesis, we found that PRP treatment significantly improved OPR in women with RIF [OR = 3.41, 95%CI (2.08, 5.60), I^2^ = 30%] (**Figure 3c**).

#### LBR

Five studies investigated LBR after different treatments in the two groups. Due to high heterogeneity (I^2^ = 75%), a random-effects model was employed for analysis. The results demonstrated that LBR was increased by PRP administration [OR=5.10, 95%CI (1.95, 13.37)] (**Figure 3d**).

#### MR

Seven studies involving 572 patients were included in the analysis. Using a random-effects model for meta-analysis, we found no significant difference in MR between groups with and without PRP [OR = 0.56, 95%CI (0.17, 1.89), I^2^ = 65%] (**Figure 3e**).

#### Preterm birth rate

Of the eleven clinical trials included in the meta-analysis, only two reported preterm birth rates. The pooled analysis revealed a significantly higher preterm birth rate in the PRP group compared to the control group [OR = 8.24, 95%CI (2.09, 32.41), I^2^ = 0%] (**Figure 3f**).

#### IR

Three studies involving 555 patients (264 in the study group and 291 in the control group) reported implantation rates (IR). As shown in **Figures 3g**, a random-effects model analysis showed no significant difference in IR between the two groups [OR = 2.15, 95%CI (0.85, 5.41), I^2^ = 77%].

#### EPR

Among the eleven clinical trials included in the meta-analysis, only two reported ectopic pregnancy rates (EPR). Pooled data analysis showed no significant difference in EPR between PRP and control groups [OR = 1.35, 95%CI (0.20, 9.11), I^2^ = 0%] (**Figure 3h**).

### Subgroup analysis

**Figure 4:**
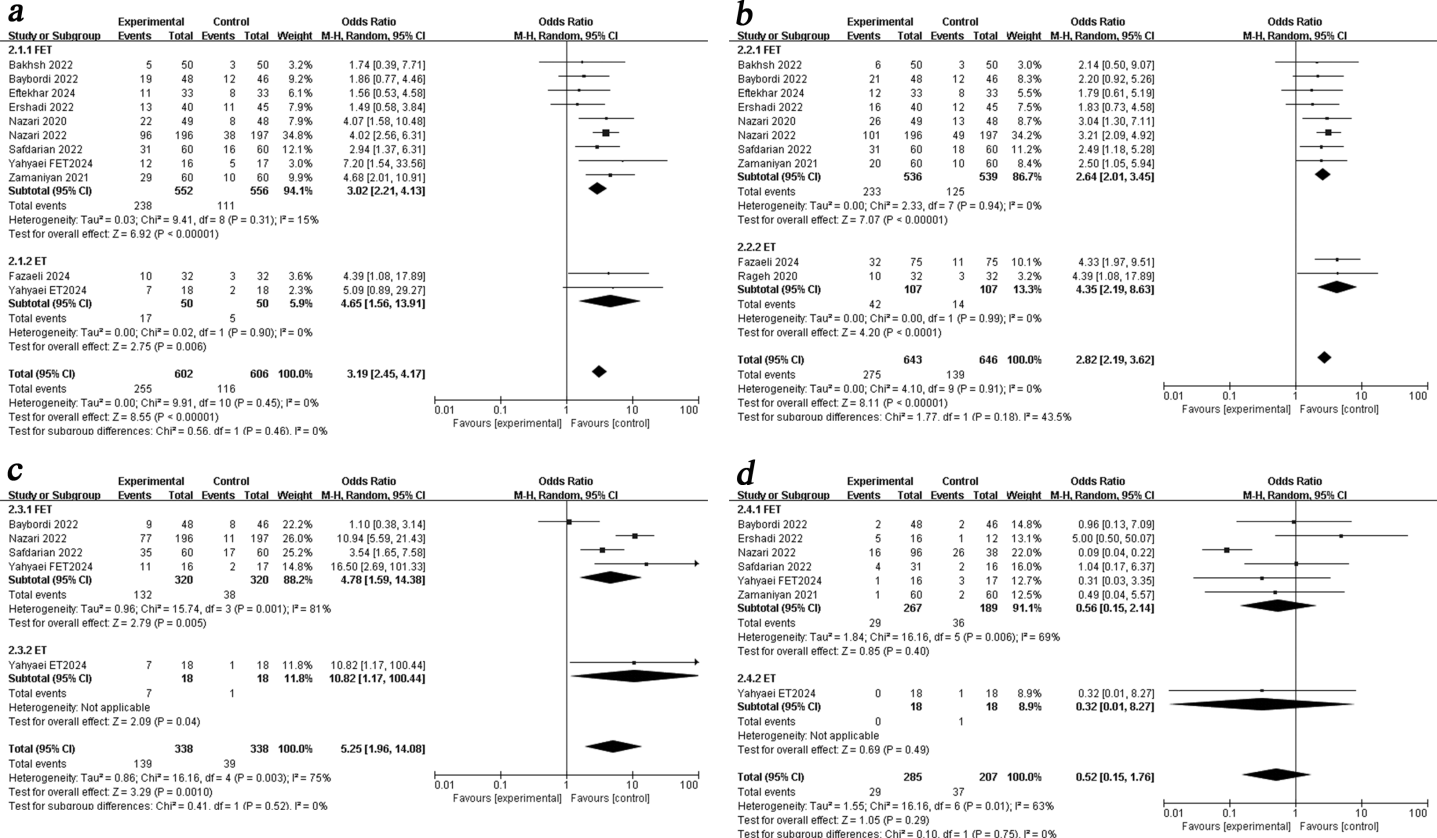
Subgroup analysis comparing the effect of PRP on pregnancy outcomes based on transfer cycle. **(a)** clinical pregnancy rate. **(b)** biochemical pregnancy rate. **(c)** live birth rate. **(d)** miscarriage rate.

We performed a subgroup analysis by embryo transfer cycle. Pooled results demonstrated that PRP administration improved CPR [fresh OR = 4.65, 95%CI (1.56, 13.91), I^2^ = 0%; frozen OR = 3.02, 95%CI (2.21, 4.13), I^2^ = 15%], BPR [fresh OR = 4.35, 95%CI (2.19, 8.63), I^2^ = 0%; frozen OR = 2.64, 95%CI (2.01, 3.45), I^2^ = 0%] and LBR [fresh OR = 10.82, 95%CI (1.17, 100.44); frozen OR = 4.78, 95%CI (1.59, 14.38), I^2^ = 81%] in both the fresh and frozen embryo transfer cycles. However, intrauterine infusion of PRP showed no beneficial effect on MR in either fresh or frozen cycles (**Figure 4**).

**Figure 5:**
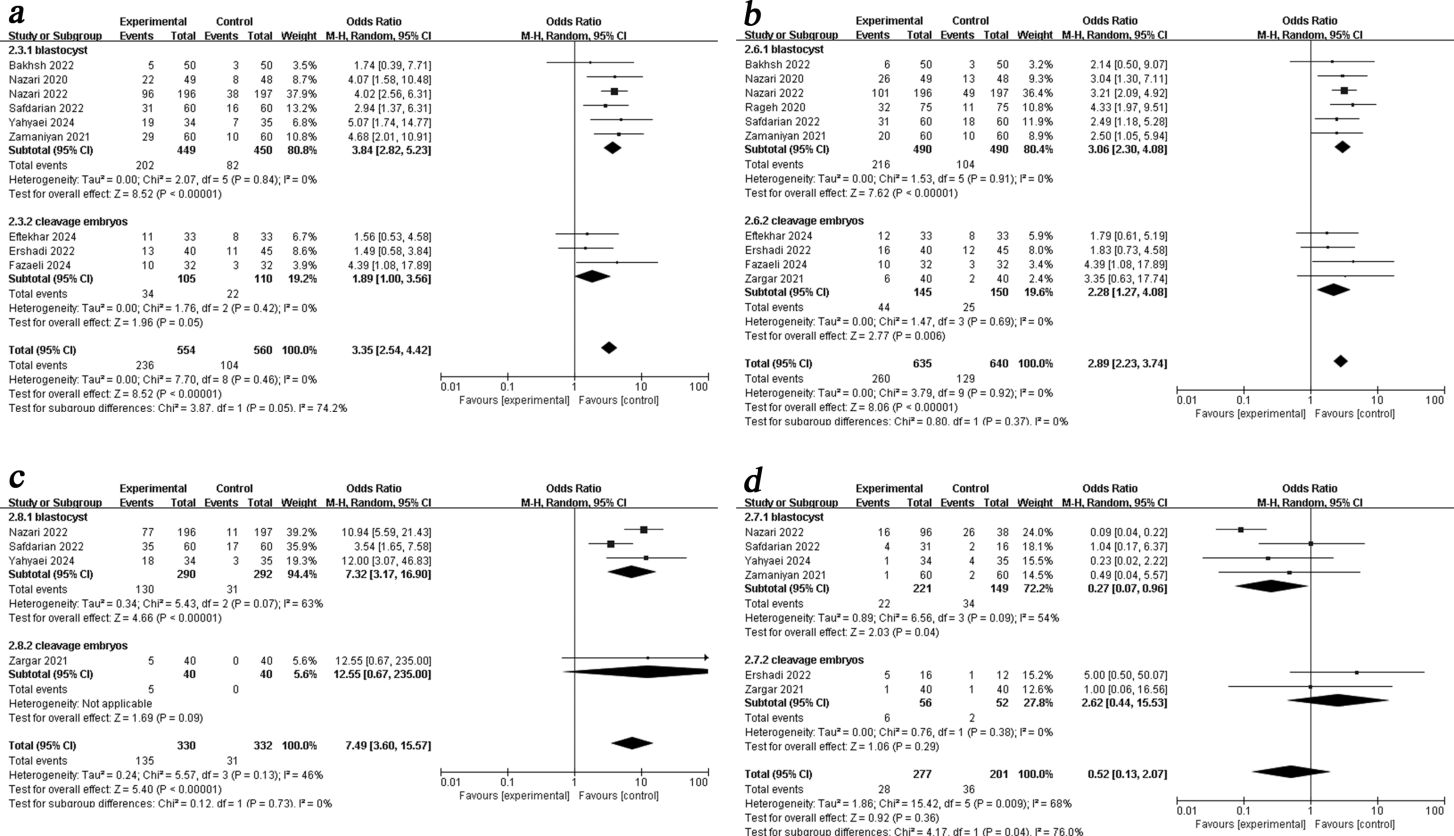
Subgroup analysis comparing the effect of PRP on pregnancy outcomes based on embryo stage. **(a)** clinical pregnancy rate. **(b)** biochemical pregnancy rate. **(c)** live birth rate. **(d)** miscarriage rate.

Subgroup analysis by transferred embryo stage revealed that PRP treatment significantly improved BPR regardless of whether blastocysts or cleavage-stage embryos were transferred [blastocyst OR = 3.06, 95%CI (2.30, 4.08), I^2^ = 0%; cleavage embryo OR = 2.28, 95%CI (1.27, 4.08), I^2^ = 0%]. Furthermore, PRP treatment increased CPR [OR = 3.84, 95%CI (2.82, 5.23), I^2^ = 0%], LBR [OR = 7.32, 95%CI (3.17, 16.90), I^2^ = 63%] and reduced MR [OR = 0.27, 95%CI (0.07, 0.96), I^2^ = 54%] in blastocyst transfers, but showed no significant effects in cleavage-stage embryo transfers (**Figure 5**).

**Figure 6:**
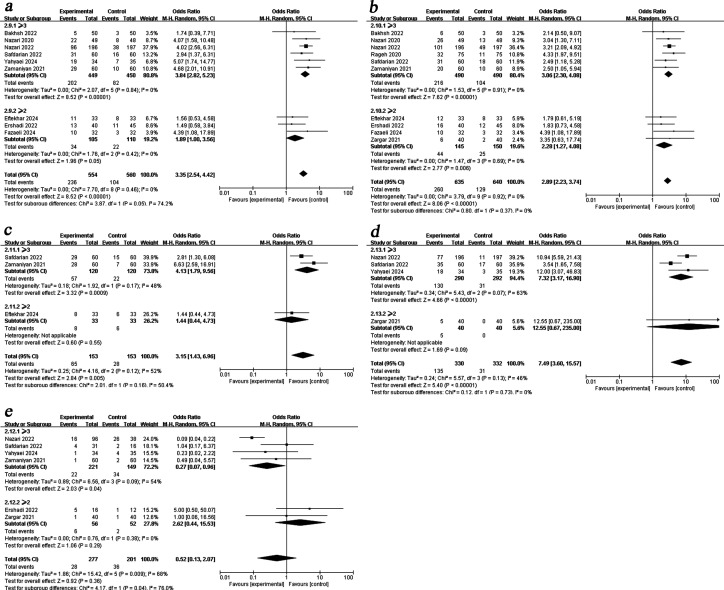
Subgroup analysis comparing the effect of PRP on pregnancy outcomes based on RIF diagnostic criteria. **(a)** clinical pregnancy rate. **(b)** biochemical pregnancy rate. **(c)**ongoing pregnancy rate. **(d)** live birth rate. **(e)** miscarriage rate.

When subgroup analysis was carried out according to the different RIF diagnostic criteria, the results showed that PRP administration associated with a higher CPR [OR = 3.84, 95%CI (2.82, 5.23), I^2^ = 0%], OPR[OR = 4.13, 95%CI (1.79, 9.56), I^2^ = 48%], LBR [OR = 7.32, 95%CI (3.17, 16.90), I^2^ = 63%] and a lower MR [OR = 0.27, 95%CI (0.07, 0.96), I^2^ = 54%] in women with ≥3 implantation failure, it did not confer the same benefit to those with a history of ≥2 failed cycles (**Figure 6**).

In the studies included in our analysis, all patients had an endometrial thickness greater than 7mm. Due to the lack of relevant primary studies, we were unable to perform a subgroup analysis based on endometrial thickness to assess the effect of PRP treatment.

### Sensitivity analysis

One study was judged to be at high risk of bias due to lack of participant blinding (30). We conducted sensitivity analysis to assess its influence on the synthesized outcomes. The pooled OR and 95% CI remained similar after excluding this study [CPR OR = 3.05, 95%CI (2.32, 4.02), I^2^ = 5%; BPR OR = 2.87, 95%CI (2.22, 3.71), I^2^ = 0%; OPR OR = 2.56, 95%CI (1.41, 4.63), I^2^ = 0%; MR OR = 0.59, 95%CI (0.15, 2.35), I^2^=70%], indicating no significant effect on overall pregnancy outcomes (See **Supplementary Figure 1**).

### Assessment of heterogeneity

Although several expert consensuses or standards for PRP preparation have been issued for specific fields or regions in recent years. Nevertheless, an internationally unified, interdisciplinary consensus has yet to be established, and heterogeneity in PRP preparation methods persists across different studies. This systematic review summarized the PRP preparation protocols of each included study (see **Table 2**). Nearly all studies employed a two-step centrifugation method for PRP preparation, and the platelet concentrations in PRP were generally comparable. However, the centrifugation speeds and durations varied across studies, which may lead to differences in leukocyte content and growth factor profiles in PRP. Nevertheless, the majority of studies did not provide corresponding data, nor did they specify whether PRP was activated, precluding the evaluation and analysis of heterogeneity.

**Table 2:** Information of PRP preparation.

Author /Year	Preparation protocol	Platelet concentration	Leukocyte content	Activation method
Nazari 2020 (28)	two-step centrifuge process (1200 rpm for 10 min; 3300 rpm for 5 min)	4-5 times higher in concentration than circulating blood	not mentioned	no inactivation step
Rageh 2020 (29)	two-step centrifuge process (1200 rpm for 12 min; 3300 rpm for 7 min)	4-5 times more than peripheral blood	not mentioned	not mentioned
Zamaniyan 2021 (30)	two times centrifuge course (1200 rpm for 12 min; 3300 rpm for 7 min)	4-7 times more than peripheral blood	not mentioned	not mentioned
Bakhsh 2022 (31)	two-step centrifuging process (1400 rpm for 10 min; 3500 rpm for 6 min)	4-5 times higher than its amount in the peripheral blood	not mentioned	not mentioned
Baybordi 2022 (32)	not mentioned	not mentioned	not mentioned	not mentioned
Ershadi 2022 (24)	two stages of the centrifugation process (1200 rpm for 12 min; 3300 rpm for 7 min)	4 or 5 times more platelets than the intravenous blood	not mentioned	not mentioned
Nazari 2022 (33)	two-step centrifuge process (1200 rpm for 12 min; 3300 rpm for 5 min)	4-5 times more than peripheral blood	not mentioned	not mentioned
Safdarian 2022 (34)	two-step centrifuge process (1600 rpm for 10 min; 3500 rpm for 6 min)	4-5 times higher than the basal blood sample	2000 lymphocyte/µL	not mentioned
Eftekhar 2024 (35)	two-step centrifuge process (1600 g for 10 min; 3500 g for 5 min)	concentration of four to five times the platelet compared to expect	not mentioned	not mentioned
Fazaeli 2024 (36)	using a commercial kit, two-step centrifuge process (1700 rpm for 10-12 min; 3800 rpm for 7 min)	4-5 times more platelets than peripheral blood	not mentioned	not mentioned
Mehrafza 2024 (37)	two steps centrifuge process (1200 rpm for 10 min; 3300 rpm for 5 min)	4-5 times the normal platelet concentration	not mentioned	not mentioned
Yahyaei 2024 (38)	using the ROOYAGEN kit, two-step centrifuge process (1800 rpm for 10 min; 3500 rpm for 6 min)	4-6 times more concentrated than the baseline platelet	not mentioned	not mentioned
Zargar 2021 (46)	using the Fertilize Lympho-PRP kit, two-step centrifuge process (12000X g for 10 min; 12000X g for 10 min)	not mentioned	not mentioned	not mentioned

### Publication bias analysis

For publication bias assessment, both funnel plot symmetry and Egger’s regression test suggested a low probability of publication bias (**Supplementary Figure 2**).

## Discussion

We conducted a systematic review and meta-analysis to evaluate the efficacy of PRP in women with RIF. Twelve RCTs provided sufficient quantitative data for statistical pooling. Our meta-analysis demonstrated that intrauterine PRP infusion may increase clinical pregnancy, biochemical pregnancy, ongoing pregnancy and live birth rates in RIF patients.

Multiple studies have identified increased endometrial thickness as a critical factor implantation and pregnancy, potentially contributing to higher pregnancy rates. The role of PRP in improving pregnancy outcomes was believed to be attribute to its effect on endometrial thickness since the study conducted by Chang et al. (18). However, this remains questionable. Enatsu et al. reported improved pregnancy rates following intrauterine PRP infusion in RIF patients regardless of endometrium thickness, despite showing no superior effect on endometrial thickness compared to conventional treatments (23). The present review intended to perform subgroup analyses stratified by endometrial thickness (<7mm vs. ≥7mm) to explore whether enhanced endometrial thickness mediates the therapeutic effect of PRP on pregnancy outcomes in RIF. However, as all included studies enrolled patients with an endometrial thickness exceeding 7mm and failed to report pre- and post-infusion thickness measurements, such an analysis was not feasible. Therefore, despite the possible favorable outcomes observed in the overall RIF population, the generalizability of these findings to RIF patients with thin endometrium is limited and warrants further investigation.

However, PRP administration failed to reduce miscarriage rates. We estimated that these outcomes may result from the single-dose PRP protocol. As the therapeutic effects diminish over time, patients with suboptimal endometrial receptivity may experience compromised pregnancy maintenance, potentially leading to miscarriage. Moreover, a normal endometrium possesses selective mechanisms to screen out low-quality embryos (e.g. those with chromosomal abnormalities). PRP administration may enhance endometrial receptivity, potentially enabling implantation of developmentally compromised embryos. Considering their diminished developmental potential, miscarriage may ultimately occur. However, the included studies did not report information on embryo ploidy screening. Future, more comprehensive and rigorously designed RCTs that include only euploid embryos are warranted to eliminate the confounding factor of embryo quality, thereby facilitating a more accurate analysis of the therapeutic effects of PRP and its impact on pregnancy outcomes.

Furthermore, it is important to note that the meta-analysis showed a statistically significant increase in preterm birth rates following PRP intervention, a finding based on data from only two of the included studies. In the study by Yahyaei (38), the incidence of preterm birth was significantly higher in the PRP group compared with the control group, with this increase observed primarily in FET cycles. Notably, pregnancy complications—specifically gestational hypertension (GH) and gestational diabetes mellitus (GDM) —occurred exclusively in the PRP group and were confined to FET cycles. Given the association between these complications and an elevated risk of preterm delivery (39), it is hypothesized that the heightened preterm birth rate in the PRP group may be partly mediated by these complications. However, the relationship between PRP treatment and pregnancy complications remains unclear. Studies have reported enhanced platelet activation in patients with preeclampsia, leading to increased release of inflammatory mediators, which may contribute to endothelial dysfunction and the pathogenesis of hypertension (40). PRP itself constitutes a concentrate of supraphysiological platelet levels, and exogenous intrauterine PRP infusion may mimic or amplify the platelet activation effects observed in this pathological condition. Consequently, a biologically plausible rationale exists for a potential increase in the risk of gestational hypertension following PRP treatment. Future randomized controlled trials (RCTs) are warranted to systematically assess perinatal outcomes—including pregnancy complications and preterm birth—in order to comprehensively evaluate the safety profile of PRP therapy. If PRP-treated pregnancies are associated with a higher risk of GDM and GH, intensified screening for these complications is recommended in patients who conceive following PRP administration—particularly those undergoing FET cycles—to enable early intervention and mitigate the risk of preterm birth. In addition, given that up to 56.6% of RIF patients exhibit a Th1-dominant endometrial immune profile (41), and considering that PRP contains both pro- and anti-inflammatory cytokines (42), it is theoretically plausible that PRP administration could exacerbate pre-existing immune dysregulation in RIF, potentially contributing to adverse pregnancy outcomes such as preterm birth. However, this hypothesis remains speculative and requires validation through studies that assess endometrial immune profiling before and after PRP intervention. In conclusion, the finding of an elevated preterm birth rate in the PRP group, albeit derived from only two studies, raises significant safety concerns and underscores the need for caution when considering PRP therapy in patients with RIF. These observations highlight an urgent requirement for future randomized controlled trials to systematically report obstetric and long-term neonatal outcomes.

Over the last two decades, endometrial injury has been studied as a potential intervention to improve implantation rates and reduce the incidence of implantation failure in IVF cycles. Many studies have reported that, compared with the control group, the endometrial injury group may have a higher CPR and LBR. During intrauterine PRP infusion, the insertion and removal of catheters are required to complete the treatment, which may cause endometrial injury. However, nearly all the studies included in our meta-analysis employed blank controls, making it impossible to assess the effect of mechanical manipulation of the uterine cavity on the pregnancy outcomes. A previous meta-analysis demonstrated that endometrial injury could improve CPR and LBR in patients with at least one failed IVF cycle. However, when analyzing only studies involving RIF patients, the difference was not statistically significant (43). This suggests that the beneficial effect of PRP on pregnancy outcomes in RIF patients may be independent of mechanical injury. Nevertheless, additional research is required. It is recommended that future randomized controlled trials employ a sham control arm—for instance, cervical catheter insertion without infusion—to effectively isolate the biological effects attributable to PRP.

There remains a high degree of variability and discrepancy exists regarding the diagnostic criteria for recurrent implantation failure (RIF) in clinical practice. Currently, published studies have primarily employed either a threshold of ≥2 or ≥3 failed embryo transfers to diagnose this condition. Importantly, the application of disparate diagnostic criteria may significantly impact the reported effectiveness of therapeutic strategies. In light of this, to address this heterogeneity and to better define the optimal target population for treatment, the present systematic review conducted a subgroup analysis stratified by the RIF diagnostic criteria used in the included studies. The findings revealed that the therapeutic benefits of PRP —including improvements in CPR and LBR, as well as a reduction in MR—were evident only in studies adopting the more stringent diagnostic threshold of ≥3 failed embryo transfers. These findings raise the possibility that the therapeutic benefits of PRP may be related to the presence of more advanced or cumulative endometrial pathology—a profile that is more likely to be identified by stricter RIF diagnostic thresholds. Conversely, inclusion of patients with only two prior failures may introduce a higher proportion of individuals whose prior implantation failures were attributable to stochastic factors rather than true pathological RIF, thereby potentially attenuating the observed treatment effect. This underscores the importance of adopting standardized, evidence-based RIF diagnostic criteria—such as the ESHRE-recommended threshold of >60% predicted implantation failure probability (44)—in future trials to ensure appropriate patient selection and more accurate assessment of PRP efficacy.

While both fresh and frozen embryo transfer cycles are commonly employed in IVF, their endometrial environments are distinct. Growing evidence indicates that endometrial receptivity is adversely affected by the supraphysiological hormonal environment in controlled ovarian stimulation (COS) cycles (45). The current systematic review and meta-analysis sought to determine whether the therapeutic benefit of PRP for patients with RIF differs according to embryo transfer protocol, through a subgroup analysis comparing outcomes in fresh versus frozen cycles. The present findings revealed improved pregnancy and live birth rates following PRP administration in both fresh and frozen embryo transfer cycles, providing preliminary evidence of potential efficacy across these two clinical contexts. Further research with adequately powered sample sizes is needed to validate these findings and to determine the conditions under which PRP may confer optimal benefit.

In addition, subgroup analyses were performed according to embryo transfer stage. The results indicated that PRP treatment was associated with significant improvements in BPR in RIF patients regardless of transfer stage. However, the beneficial effects on CPR and MR were confined to blastocyst transfer cycles. This pattern of findings points to a potential advantage of blastocyst-stage transfer in the context of PRP treatment for RIF. However, due to insufficient data, a pooled analysis of preterm birth rates could not be performed in the subgroup analyses. Given that blastocyst transfer has been independently associated with an increased risk of preterm birth (47), the observation of elevated preterm birth rates in the PRP group in the present study therefore raises a noteworthy concern. This finding underscores the importance of careful consideration when selecting treatment protocols and embryo transfer strategies, and highlights the need for further rigorous investigations to clarify these risks.

## Limitations

Several limitations should be considered when interpreting the results of this meta-analysis. First, heterogeneity existed among the included studies, particularly regarding PRP preparation. Although the centrifugation protocols varied across studies—with differences in both speed and duration—the platelet concentrations in the prepared PRP were generally comparable. However, these methodological discrepancies may translate into meaningful differences in leukocyte content and growth factor profiles (48). Leukocytes may play a dual role in PRP therapy—potentially contributing to sustained growth factor release while also introducing pro-inflammatory elements that could counteract beneficial effects (49). Furthermore, variations in the growth factor and cytokine profiles of PRP may lead to clinically meaningful differences in reproductive outcomes (50). Future studies should adhere to emerging recommendations for the standardized reporting of PRP composition, including platelet count, leukocyte differential, and quantification of key growth factors and cytokines, to enhance cross-study comparability and facilitate the identification of optimal PRP characteristics for reproductive applications. Second, the validity of our meta-analysis may be undermined by the limited number of included studies, considerable methodological heterogeneity, and incomplete reporting of pregnancy outcomes. These limitations not only introduce potential bias and reduce the robustness of the pooled estimates, but also preclude a comprehensive assessment of critical safety outcomes, particularly preterm birth. Finally, the included studies did not report whether embryo euploidy testing was performed. We recommend that future studies provide more detailed methodological descriptions, particularly when comparing multiple embryo transfer approaches. Despite these limitations, our study offers a comprehensive evaluation of current evidence, guided by a prospectively registered protocol. The conclusions represent a rigorous synthesis of available data on this emerging therapy.

## Conclusions

These findings suggest a potential but inconclusive role for PRP in improving pregnancy outcomes among women with RIF, particularly in those with a history of three or more prior implantation failures. Furthermore, the use of blastocyst-stage embryo transfer may be associated with an increased likelihood of live birth and a reduced risk of miscarriage. However, given the limited safety data, further investigation is warranted to clarify the possibility that PRP treatment may be associated with an elevated risk of preterm birth. To this end, future studies should adopt standardized protocols for PRP preparation and composition reporting to enhance cross-study comparability and enable more robust evidence synthesis.

## Data Availability

The original contributions presented in the study are included in the article/**Supplementary Material**. Further inquiries can be directed to the corresponding authors.
