# Supplementary figures and images for: Effects of autologous platelet-rich plasma in recurrent implantation failure: a systematic review and meta-analysis
Source: Front Endocrinol (Lausanne). 2026 Apr 14;17:1730259. doi: 10.3389/fendo.2026.1730259 (PMC13120909; doi:10.3389/fendo.2026.1730259)

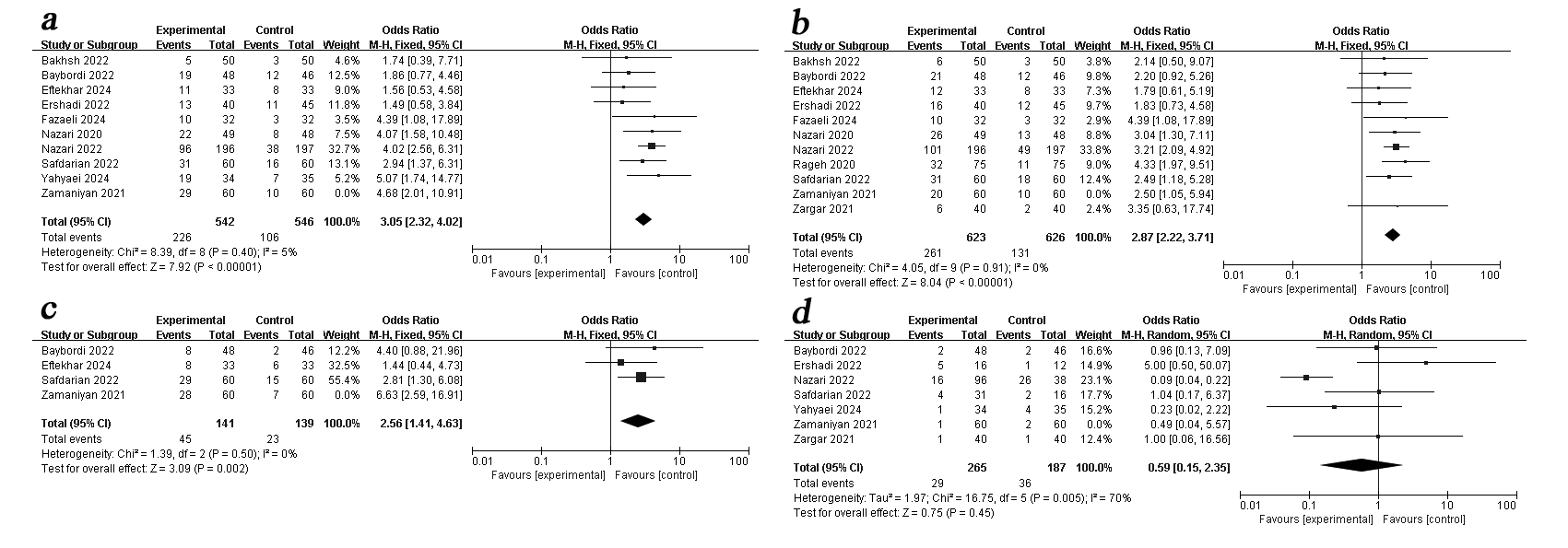

Supplement: Supplementary file 1 [file Image1.tif]

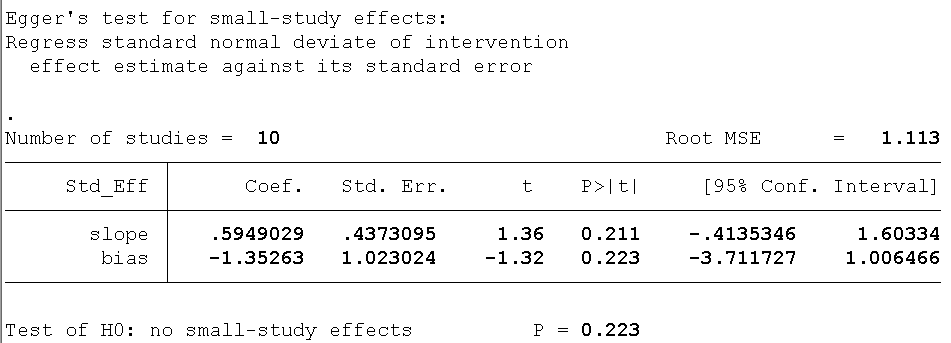

Supplement: Supplementary file 2 [file Image2.tif]
